# Arrest of Cytoplasmic Streaming Induces Algal Proliferation in Green Paramecia

**DOI:** 10.1371/journal.pone.0001352

**Published:** 2007-12-26

**Authors:** Toshiyuki Takahashi, Yohji Shirai, Toshikazu Kosaka, Hiroshi Hosoya

**Affiliations:** Graduate School of Biological Science, Hiroshima University, Higashi-Hiroshima, Hiroshima, Japan; Ordway Research Institute, United States of America

## Abstract

A green ciliate *Paramecium bursaria*, bearing several hundreds of endosymbiotic algae, demonstrates rotational microtubule-based cytoplasmic streaming, in which cytoplasmic granules and endosymbiotic algae flow in a constant direction. However, its physiological significance is still unknown. We investigated physiological roles of cytoplasmic streaming in *P. bursaria* through host cell cycle using video-microscopy. Here, we found that cytoplasmic streaming was arrested in dividing green paramecia and the endosymbiotic algae proliferated only during the arrest of cytoplasmic streaming. Interestingly, arrest of cytoplasmic streaming with pressure or a microtubule drug also induced proliferation of endosymbiotic algae independently of host cell cycle. Thus, cytoplasmic streaming may control the algal proliferation in *P. bursaria*. Furthermore, confocal microscopic observation revealed that a division septum was formed in the constricted area of a dividing paramecium, producing arrest of cytoplasmic streaming. This is a first report to suggest that cytoplasmic streaming controls proliferation of eukaryotic cells.

## Introduction

Active movement of organelles called as cytoplasmic streaming has been observed in various kinds of cells such as green algae *Nitella* and *Chara*, embryos of Caenorhabditis elegans, amoeba and ciliates [1]–[4]. Cytoplasmic streaming aids in the delivery of nutrients, metabolites, and genetic information to all parts of larger plant cells. The transport of mitochondria is vital for the development and maintenance of axons in the nervous system and for the establishment of embryonic polarity in the early embryos of *C. elegans*. Especially, in plant and amoeboid cells, successful biochemical and biophysical approaches to studying actin/myosin system have extensively promoted the understanding of the molecular mechanism underlying cytoplasmic streaming.

A ciliate *Paramecium bursaria* harbors several hundreds of endosymbionts like *Chlorella* in the cytoplasm [5]. It is well known that *P. bursaria* demonstrates rotational cytoplasmic streaming [3], in which some cytoplasmic granules and endosymbionts flow in a constant direction. Although it has been elucidated that the cytoplasmic streaming in green paramecia is microtubule- but not actin-based [3], its physiological significance is still unknown.

Here, we found that no cytoplasmic streaming was observed in dividing paramecia, while endosymbionts were proliferating in host cells. Several experiments revealed that arrest of cytoplasmic streaming induced proliferation of endosymbionts in interphase paramecia. These data suggest that cytoplasmic streaming controls algal proliferation in green paramecia. This is the first report to show that cytoplasmic streaming works as a signal to control proliferation of eukaryotic cells.

## Materials and Methods

### Strain and culture condition of *Paramecium bursaria*

*Paramecium bursaria* syngen 1 (BWK-4, mating type IV; KN-21, III) were used in this study. Stocks BWK-4 and KN-21 were collected from the Lake Biwa (Shiga prefecture, Japan) and the river Kino-kawa (Wakayama prefecture, Japan), respectively. A symbiotic algae-free strain (BWKw-4) was produced by a herbicide paraquat [5]. Paramecia were cultured in lettuce infusion containing the bacterium *Klebsiella pneumoniae* as food under a LD cycle (12 h light/12 h dark) at *ca.* 1500 lux of natural white fluorescent light and 23°C [6].

### Strain and culture condition of exsymbiotic algae isolated from *P. bursaria*

Cloned exsymbiotic algae (SA-1 and SA-3) isolated from *P. bursaria* were obtained as described previously [7]. Each algal strain was cultured in a CA medium under constant light at *ca.* 1500 lux of natural white fluorescent light and 24°C [7].

### Observation of cytoplasmic streaming

To observe cytoplasmic streaming in *P. bursaria*, carmine powder was added into a paramecium culture and the flow of carmine particles in a paramecium cell was observed with video-microscopy. In this study, paramecia showing cytoplasmic streaming and without streaming were expressed as CS+ and CS-, respectively.

### Measurement of the number of endosymbiotic algae per paramecium

A paramecium was squashed and the total number of the endosymbionts was counted with light microscopy [8].

### Condition to arrest cytoplasmic streaming in paramecia by pressure

Cytoplasmic streaming in *P. bursaria* at interphase was artificially arrested for 2 hr in a moisture chamber by pressure with a cover slip-glass slide spacing of 17 µm as described previously [3]. After giving the pressure with a cover slip-glass slide, living paramecia were collected and used to measure the number of endosymbionts.

### Condition to arrest cytoplasmic streaming in paramecia by addition of nocodazole

It has been reported that cytoplasmic streaming in *P. bursaria* is arrested by nocodazole [3]. In this study, nocodazole was used to arrest cytoplasmic streaming in *P. bursaria*. To investigate roles of cytoplasmic streaming in *P. bursaria*, nocodazole in DMSO was added into a paramecium culture (final 1% DMSO) [3]. After the treatment with nocodazole (final concentration ≥3 µg/ml nocodazole) for 48 hr under constant light condition, living paramecia were collected and used to measure the number of endosymbionts.

### Preparation of specimens for fluorescence microscopy and confocal laser scanning microscopy

Each dividing paramecium was harvested using a pipette and placed on a coverslip. The cells were fixed with methanol for 6 hr at −20°C. The fixed paramecia were washed twice with PBS. The cells were mounted in FluoroGuard™ (Bio-Rad) containing 4′, 6-diamidino-2-phenylindole (DAPI: Nakalai Tesque Inc.) for DNA staining. The specimen prepared was observed under both a Nomarski differential interference contrast (DIC) and a fluorescence microscope (Optiphot; Nikon, Tokyo, Japan) equipped with a digital camera (COOLPIX 900; Nikon, Tokyo, Japan) and a confocal laser scanning microscope (LSM 410, Zeiss Jena, Germany). In LSM observation, serial fluorescence sections of 1 µm were obtained and final images were compiled with Adobe Photoshop software.

## Results

### Cytoplasmic Streaming in a Dividing Green Paramecium

**Figure 1:**
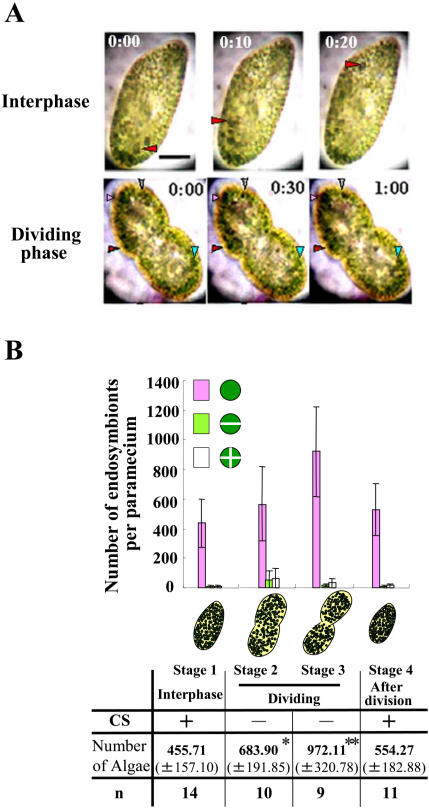
(A) Cytoplasmic streaming in *P. bursaria.* Upper (10-second intervals) and Lower panels (30-second intervals); *P. bursaria* having carmine particles (arrowheads) in the cytoplasm at interphase (stage 1) and dividing phase (stage 2), respectively. Scale bar, 20 µm. (B) A relation between cytoplasmic streaming and the number of endosymbionts. A bar graph shows the number of endosymbionts (±S.D.) at each phase of host cell cycle (stage 1–4). The endosymbionts were classified into a unicellular cell (purple), 2 (green) and 4 autospores (white). A table shows whether cytoplasmic streaming occurs or not, and the total number of endosymbionts. * and ** mean statistical differences with Student's *t*-test, *P<0.005 and **P<0.001.

Cytoplasmic streaming in *Paramecium bursaria* (green and algae-free) was observed *in vivo*. *P. bursaria* at interphase demonstrates dynamically rotational microtubule-based cytoplasmic streaming [3], in which some cytoplasmic granules and endosymbionts flow in a constant direction (Fig. 1A, upper; Movie S1). Interestingly, we found that dividing host cells almost arrested cytoplasmic streaming (Fig. 1A, lower; Movie S1). The arrest of cytoplasmic streaming continued until about 30 min after host cell division (data not shown). When a paramecium initiated cytokinesis (Stage 2, Fig. 1B), endosymbionts also initiated cytokinesis, producing 2–4 autospores. In Stage 3, the total number of endosymbionts increased twice as much as that at interphase (Stage 1). When a paramecium completed cell division and cytoplasmic streaming recovered (Stage 4), the number of endosymbionts returned almost the same as that at interphase.

### Effect of Artificial Arrest of Cytoplasmic Streaming in *P. bursaria* on Endosymbionts

**Figure 2:**
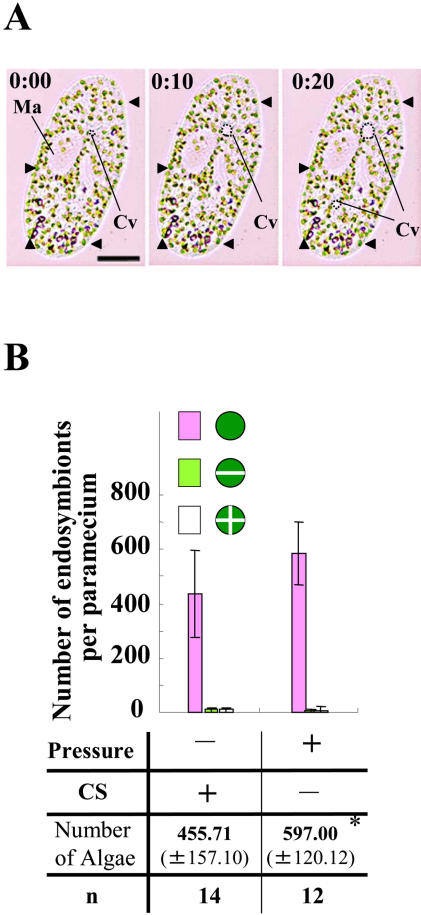
(A) Arrest of cytoplasmic streaming in *P. bursaria* by pressure (10-second intervals). *P. bursaria* at interphase arresting cytoplasmic streaming under pressure. Ma and Cv indicate macronucleus and contractile vacuole (broken lines), respectively. Scale bar, 20 µm. (B) Cytoplasmic streaming-dependent increase of the number of endosymbionts. A bar graph shows the number of endosymbionts (±S.D.) under pressure or non-pressure (control). The control value is the number of endosymbionts at intephase (stage 1) described in Fig. 1B. * means a statistical difference *P*<0.02.

We have shown that cytoplasmic streaming in *P. bursaria* is arrested both by pressure with glass coverslip spacing and by addition of microtubule drug nocodazole [3]. To further investigate roles of cytoplasmic streaming in *P. bursaria*, we determined the number of endosymbionts under pressure with a coverslip. As shown in Fig. 2A, the pressure arrested cytoplasmic streaming in *P. bursaria* at interphase, although the contractile vacuole (Cv) kept moving. Interestingly, endosymbionts started increasing within 2 hrs after initiating arrest of cytoplasmic streaming (Fig. 2B).

**Figure 3:**
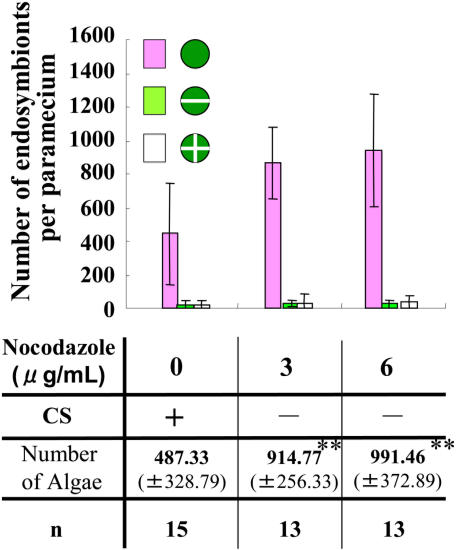
The bar graph shows the number of endosymbionts (±S.D.) after treatment with nocodazole or with 1% DMSO. ** means a statistical difference *P*<0.001.

It has been known that a microtubule drug nocodazole arrests cytoplasmic streaming in green paramecia [3]. When nocodazole was added to arrest cytoplasmic streaming in *P. bursaria* (Movie S2), the number of endosymbionts also increased (Fig. 3). The paramecia treated with 6 µg/ml nocodazole had larger numbers of endosymbionts than paramecia treated with 3 µg/ml nocodazole (Fig. 3). The concentrations of nocodazole used completely arrested the growth of host paramecia (data not shown), while nocodazole used did not arrest growth of endosymbionts isolated from *P. bursaria* (Fig. S1). Thus, the results obtained show that arrest of cytoplasmic streaming induces proliferation of endosymbionts independently of host cell cycle.

### Movement and Localization of Endosymbionts in Dividing Paramecium

**Figure 4:**
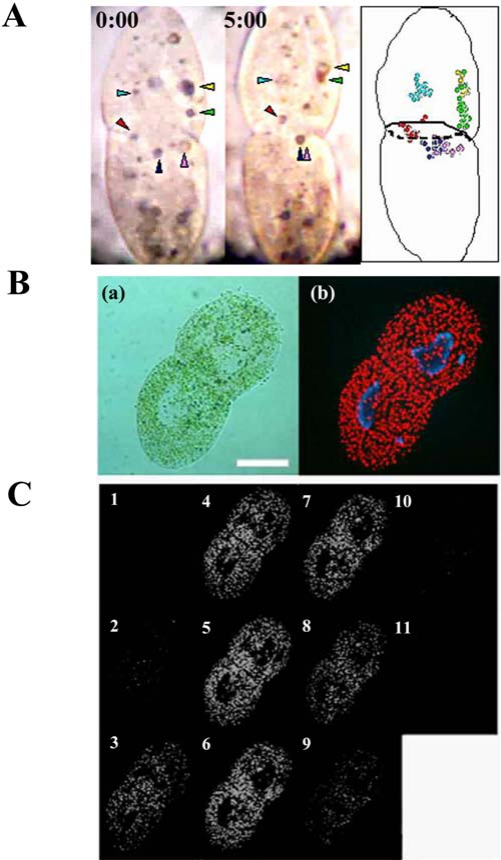
(A) Movement of carmine particles near the division plane in algae-free *P. bursaria* at dividing phase. An algae-free *P. bursaria* having carmine particles (arrowheads) was monitored using video-microscopy (Left and Middle panel: 0 min and 5 min after initiating the observation, respectively). The right panel is a schematic diagram of movements of carmine particles. (B) *P. bursaria* at dividing phase. Microphotographs indicate a differential interference contrast image (a) and the corresponding conventional fluorescence image (b). Scale bar, 50 µm. (C) Serial fluorescence sections of dividing paramecia obtained by confocal microscope. Endosymbionts were eliminated from the division plane in *P. bursaria* at dividing phase.

To investigate the mechanism of arrest of cytoplasmic streaming, we monitored the movements of carmine particles near the constricted area of dividing algae-free *P. bursaria* ingesting carmine powder. As shown in Fig. 4A and Movie S3, the particles did not rotationally move in the whole cell. Further, time lapse images showed that the particles in one daughter cell have not been transferred to another cell (Fig. 4A, right). These data suggest that dividing *P. bursaria* forms a specific structure, like a plant cell plate, at the constricted area to arrest cytoplasmic streaming. DIC image of a dividing green paramecium clearly showed that there is an area eliminating endosymbionts from a constricted region in dividing paramecia (Figs. 4B, panel a, and Movie S4). Fluorescent observation revealed that endosymbiotic algae were eliminated from this area in dividing paramecia (Figs. 4B, panel b). Interestingly, serial images obtained using confocal microscope revealed that the area forms a kind of division septum (Fig. 4C). From these data, we have deduced that the division septum formed at the constricted area of dividing host cell arrested the rotational cytoplasmic streaming during cytokinesis.

## Discussion

This report using video-microscopy has revealed that cytoplasmic streaming in *Paramecium bursaria* is regulated though host cell cycle. Thus, rotational cytoplasmic streaming in *P. bursaria* at interphase is almost arrested during dividing phase (Fig. 1A and Movie S1). Further, endosymbiotic algae increased only during the arrest of cytoplasmic streaming (Fig. 1B). These results suggest that *P. bursaria* controls the proliferation of endosymbionts through host cell cycle-dependent cytoplasmic streaming.

It has been elucidated that microtubule drugs such as oryzalin but not nocodazole arrest the cell division of algae [9]. On the other hand, it has been reported that cytoplasmic streaming in *P. bursaria* is microtubule-dependent and arrested by nocodazole [3]. These might be due to the cell permeability of drugs or specialized features of microtubules in algae or paramecia. In this study, nocodazole was used as a specific inhibitor to arrest only cytoplasmic streaming in *P. bursaria* but not the division of symbiotic algae (Fig. S1). Here, we observed that endosymbionts started proliferating in the interphasic paramecia treated with nocodazole, showing no cytoplasmic streaming. The paramecia treated with 6 µg/ml nocodazole had larger numbers of endosymbionts than paramecia treated with 3 µg/ml nocodazole (Fig. 3), because cytoplasmic streaming in *P. bursaria* under 6 µg/ml nocodazole was arrested earlier than under 3 µg/ml nocodazole (data not shown). The increase of number of endosymbionts was also observed in the paramecia pressed with glass coverslip, which show no cytoplasmic streaming (Fig. 2). Taken together, in every condition showing no cytoplasmic streaming, endosymbionts proliferated in host cells. From these data, we concluded that cytoplasmic streaming controls proliferation of endosymbionts in *P. bursaria*. This is the first report to show that cytoplasmic streaming is a key phenomenon to control cytokinesis of endosymbionts in host cells.

Our previous confocal microscopic observation revealed that, in paramecia treated with nocodazol, the number of cytoplasmic microtubules but not clusters of microtubules around the macronucleus was markedly decreased relative to control specimens. Further, ciliate microtubules on the cell surface hardly differed between control and nocodazol-treated cells. From these data, it is expected that the cytoplasmic microtubules would serve as a rail for cytoplasmic streaming. To confirm this, it should be elucidated whether microtubule structures are maintained or not in the cell, when cytoplasmic streaming is arrested by the compression. It has been shown that, in the presence of an inhibitor for microtubule motor protein dynein, the cytoplasmic streaming was arrested in green paramecia [3], although it was not examined whether algal proliferation was induced or not. Thus, it will be required to elucidate in details how microtubules and/or their motors control algal proliferation through arrest of cytoplasmic streaming.

Interestingly, DIC and fluorescence images of a dividing green paramecium clearly showed that there is an area eliminating endosymbionts from a constricted region in dividing paramecia (Fig. 4). These data suggest that a division septum formed at dividing phase arrests the rotational cytoplasmic streaming during cytokinesis. Further experiments to elucidate components of the division septum are required. Cytokinesis is the final event of cell division and is the process that divides one mother cell into two daughter cells. Several organisms including both prokaryotic and eukaryotic cells form division septum at cytokinesis. In general, bacteria form a division septum composed of Fts Z protein, homologous to tubulin, at cytokinesis and both mitochondria and chloroplasts originated from prokaryotic cells also form the septum composed of Fts Z [10]–[14]. Yeast form both a division septum composed of septins and a contractile ring based on actomyosin ring [15]. Plants form cell plate as a division septum at cytokinisis, while animal cells divide with only a contractile ring based on actomyosin ring and do not form a structure like a division septum [15], [16]. However, no past research with protozoa including ciliates has documented any structure like a division septum [17], [18], althouth several reports have shown a structure like a contractile ring [19], [20]. Results obtained in this study suggested that a division septum formed in the constricted area of dividing *P. bursaria* causes the arrest of cytoplasmic streaming.

Equal distribution of endosymbionts to daughter cells is also essential to maintain endosymbiosis in *P. bursaria*. It remains unclear how the delivery of endosymbionts to two daughter cells is controlled during cytokinesis of *P. bursaria*. Although this report gives a novel insight to functions of cytoplasmic streaming, it should be further determined how cytoplasmic streaming controls distribution of organelles during cell division of higher eukaryotic cells.

## Supporting Information

Figure S1 Effects of microtubule drug nocodazole on exsymbiotic algae isolated from *P. bursaria*. Exsymbiotic algae (1×104 cells/ml) were pre-incubated in CA medium for 48 hr under constant light condition. After pre-incubation of endosymbionts (arrow), nocodazole was added into the algal culture. 24 hr and 48 hr after treatment with nocodazole, the number of exsymbiotic algae was determined with a hemocytometer. The result obtained shows that nocodazole was ineffective in the growth of exsymbiotic algae.
(0.71 MB PDF)

Movie S1 Cytoplasmic streaming in *P. bursaria* having carmine particles. Cytoplasmic streaming is arrested in a dividing paramecium cell and particles locally oscillate. Note that the movie changes from a interphase paramecium (former movie: until about 30 seconds) to a dividing paramecium (latter movie).
(1.98 MB MOV)

Movie S2 Cytoplasmic streaming in *P. bursaria* at interphase was arrested in the presence of 6 µg/ml nocodazole.
(0.36 MB MOV)

Movie S3 Movements of carmine particles near a division plane in *P. bursaria* (algae-free strain) at dividing phase. Although dividing cells arrested dynamically rotational cytoplasmic streaming, the carmine particles near the division plane slightly moved. The particles have never penetrated the division plane. Note that the movie is played at 4-fold speed.
(0.96 MB MOV)

Movie S4 The arrest of cytoplasmic streaming and the gap near a division plane in *P. bursaria*. After arrest of cytoplasmic streaming in a dividing paramecium cell, endosymbionts were eliminated from the division plane. The division plane looks like a white line due to the exclusion of symbiotic green algae. Note that the movie changes from an interphase paramecium (former movie: until about 15 seconds) to a dividing paramecium (latter movie).
(0.95 MB MOV)
