# Supplementary figures and images for: Arrest of Cytoplasmic Streaming Induces Algal Proliferation in Green Paramecia
Source: PLoS One. 2007 Dec 26;2(12):e1352. doi: 10.1371/journal.pone.0001352 (PMC2131778; doi:10.1371/journal.pone.0001352)

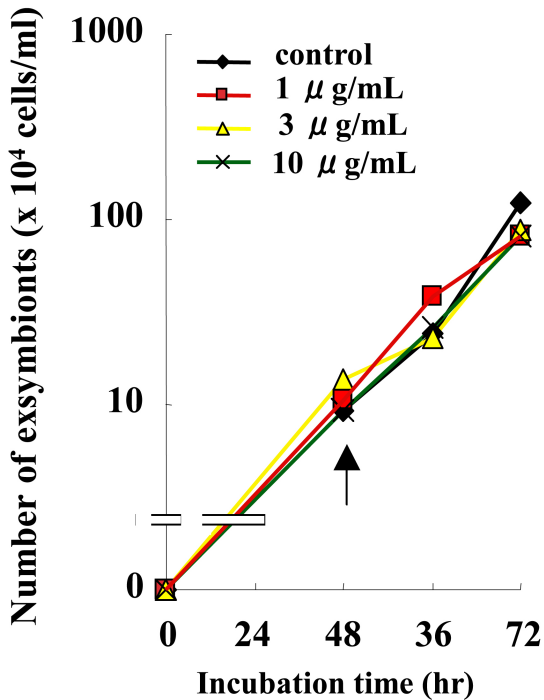

Supplement: Figure S1 — Effects of microtubule drug nocodazole on exsymbiotic algae isolated from P. bursaria. Exsymbiotic algae (1×10^4^ cells/ml) were pre-incubated in CA medium for 48 hr under constant light condition. After pre-incubation of endosymbionts (arrow), nocodazole was added into the algal culture. 24 hr and 48 hr after treatment with nocodazole, the number of exsymbiotic algae was determined with a hemocytometer. The result obtained shows that nocodazole was ineffective in the growth of exsymbiotic algae. (0.71 MB PDF) [file pone.0001352.s001.pdf]
